# Oral microbial dysbiosis in patients with oral cavity cancers

**DOI:** 10.1007/s00784-024-05770-8

**Published:** 2024-06-17

**Authors:** Ozge Unlu, Mehmet Demirci, Tugce Paksoy, Arzu Baygul Eden, Hasan Deniz Tansuker, Aysegul Dalmizrak, Cagdas Aktan, Firdevs Senel, Ahmet Volkan Sunter, Ozgur Yigit, Burak Omur Cakir, Alpdogan Kantarci

**Affiliations:** 1 Faculty of Medicine, Department of Medical Microbiology, Istanbul Atlas University, Istanbul, Turkey; 2 Faculty of Medicine, Department of Medical Microbiology, Kırklareli University, Kırklareli, Turkey; 3 Faculty of Dentistry, Department of Periodontology, University of Health Sciences, Istanbul, Turkey; 4 Faculty of Medicine, Department of Biostatistics, Koc University, Istanbul, Turkey; 5 Faculty of Medicine, Department of Otolaryngology, Yeditepe University, Istanbul, Turkey; 6 Faculty of Medicine, Department of Medical Biology, Balıkesir University, Balıkesir, Turkey; 7 Faculty of Medicine, Department of Medical Biology, Bandirma University, Balıkesir, Turkey; 8 Faculty of Dentistry, Department of Oral & Maxillofacial Surgery, Beykent University, Istanbul, Turkey; 9 Department of Ear, Nose and Throat Diseases, Istanbul Sisli Hamidiye Etfal Research and Training Hospital, Istanbul, Turkey; 10 Faculty of Medicine, Department of Ear, Nose and Throat Diseases, Istanbul Aydin University, Istanbul, Turkey; 11 ADA Forsyth Institute, Cambridge, MA USA; 12 School of Dental Medicine, Harvard University, Boston, MA USA

**Keywords:** Oral cavity cancers, Oral microbiota, Saliva, Periodontal disease

## Abstract

**Objectives:**

The pathogenesis of oral cavity cancers is complex. We tested the hypothesis that oral microbiota dysbiosis is associated with oral cavity cancer.

**Materials and methods:**

Patients with primary oral cavity cancer who met the inclusion and exclusion criteria were included in the study. Matching healthy individuals were recruited as controls. Data on socio-demographic and behavioral factors, self-reported periodontal measures and habits, and current dental status were collected using a structured questionnaire and periodontal chartings. In addition to self-reported oral health measures, each participant received a standard and detailed clinical examination. DNA was extracted from saliva samples from patients and healthy controls. Next-generation sequencing was performed by targeting V3-V4 gene regions of the 16 S rRNA with subsequent bioinformatic analyses.

**Results:**

Patients with oral cavity cancers had a lower quality of oral health than healthy controls. *Proteobacteria, Aggregatibacter, Haemophilus*, and *Neisseria* decreased, while *Firmicutes, Bacteroidetes, Actinobacteria, Lactobacillus, Gemella*, and *Fusobacteria* increased in oral cancer patients. At the species level, *C. durum, L. umeaens, N. subflava, A. massiliensis*, and *V. dispar* were significantly lower, while *G. haemolysans* was significantly increased (*p* < 0.05). Major periodontopathogens associated with periodontal disease (*P. gingivalis* and *F.nucleatum*) increased 6.5- and 2.8-fold, respectively.

**Conclusion:**

These data suggested that patients with oral cancer had worse oral health conditions and a distinct oral microbiome composition that is affected by personal daily habits and may be associated with the pathogenicity of the disease and interspecies interactions.

**Clinical relevance:**

This paper demonstrates the link between oral bacteria and oral cancers, identifying mechanistic interactions between species of oral microbiome.

**Supplementary Information:**

The online version contains supplementary material available at 10.1007/s00784-024-05770-8.

## Introduction

Oral cavity cancers are the most prevalent head and neck cancers, causing approximately 188.438 deaths annually, which makes it the cancer with the fifteenth highest mortality rate, presenting a serious health concern on a global scale [1]. Oral malignancy is multifaceted and frequently linked to smoking, alcohol consumption, chewing areca nuts, eating habits, immunodeficiency, HPV infection, and genetic factors [2, 3]. In addition, poor oral hygiene, poor nutrition, and wearing dental prostheses were associated with an increased risk of oral cancer [4–6].

Microbial factors can be contributory factors in oral cancer pathogenesis. Specific microbial species in the human microbiome may cause persistent inflammation in distinct cancer types [7]. The oral cavity has the second-highest density of microorganisms, with about 700 species, including bacteria, fungi, viruses, archaea, and protozoans [8, 9]. Since bacterial dysbiosis is associated with an increased risk of other systemic diseases, altered microbiota at tumor locations may suggest an involvement of oral bacteria in the development and progression of malignancy [3, 9]. Indeed, certain cancer types, such as gastrointestinal cancer, esophageal cancer, oral cancer, and pancreatic cancer, have been linked to several periodontal pathogens, including *Porphyromonas gingivalis, Tannerella forsythia*, and *Prevotella intermedia* [10, 11], suggesting that periodontal disease-associated dysbiosis may pose a risk for oral cancer pathogenesis. Primary tumors linked to the oral microbiota have been detected in the oral cavity and the esophagus, stomach, pancreas, and colon, suggesting that dysbiotic changes in the oral cavity may increase the risk for non-oral pathologies.

While bacteria can link oral diseases and oral cavity cancers through several pathways, there is currently a very limited understanding of the role of oral microbiotas in oral cavity cancer pathogenesis [11]. Since cancer staging is crucial in oncotherapeutic planning, early detection may alter the course of treatment, survival rates, and quality of life. However, no reliable biomarkers for early diagnosing oral cavity malignancies are currently available, where detection of oral microbial shifts may also serve as predictors of oncopathogenesis [12]. Thus, we tested the hypothesis that oral microbiota dysbiosis is associated with oral cavity cancer and that specific species can be used as disease markers.

## Materials and methods

### Study design and participants

This study was planned as a case-control study; it was approved by the Ethical Committee of the Istanbul Research and Training Hospital (2017/11/09-1109). Samples were collected after obtaining informed consent; the trial was carried out in conformity with the Declaration of Helsinki. Patients were recruited from the otorhinolaryngology clinics of Bağcılar Research and Training Hospital and Istanbul Research and Training Hospital. Oral cavity cancer diagnosis was confirmed by histopathology, and the lesions were classified using the TNM system [13]. All patients were recently diagnosed cases and none of them received any treatment. The exclusion criteria were complete edentulism, surgical operation on salivary glands, history of autoimmune disease or systemic disease, pregnancy, history of radiotherapy or chemotherapy, fixed orthodontic treatment, antibiotic, anti-inflammatory, and anticoagulant therapy or probiotic use for the past 4 weeks, non-adults (< 18 ages), active viral, bacterial, or fungal infections, and HPV positivity (tested for HPV using a commercial HPV real-time PCR kit). Out of more than 100 patients recruited, 10 patients with primary oral cavity cancers (6 men and 4 women) met the inclusion and exclusion criteria in clinical characterization, saliva sampling, and microbial analyses. Twelve systemically healthy individuals with no cancer (6 men and 6 women) were recruited as controls.

### Demographic and dental characterization of study cohorts

To characterize the study cohorts, data on socio-demographic and behavioral factors, self-reported periodontal measures and habits, and current dental status were collected using a structured questionnaire and periodontal chartings. Socio-demographic variables included age (years), gender (male/female), educational level (≤ 8 years vs.> 8 years) [14], having comorbidities (yes vs. no), familial history of cancer (yes vs. no), history of smoking (yes vs. no), current smoking status (yes vs. no), and alcohol consumption (yes vs. no) [15]. Self-reported measures of oral health behavior included tooth-brushing habits (yes vs. no), use of dental floss/interdental brush (yes vs. no), and dental check-ups (yes vs. no). We also asked the participants whether they had a history of periodontal diseases and dental prostheses [16]. Self-reported periodontal disease was assessed by gingival bleeding, swelling, and redness [17, 18]. Self-rated oral health was assessed using a single-item question with ordinal response options [16].

### Clinical examination of study individuals

In addition to self-reported oral health measures, each participant received a standard and detailed clinical examination. Periodontal examination was performed by a single experienced and calibrated examiner (T.P.). Periodontal parameters included probing depth (PD), clinical attachment level (CAL), bleeding on probing (BOP), gingival recession (GR), plaque index (PI), and gingival index (GI) [19, 20]. Clinical parameters were evaluated in all teeth, excluding third molars. PI and GI were recorded at four sites, while PD and CAL were measured at six sites per tooth (buccal, mesiobuccal, distobuccal, lingual, mesiolingual, and distolingual). BOP was measured by the presence or absence of bleeding 10 s after probing. All measurements were performed using a calibrated millimeter periodontal probe (PCP15; Hu-Friedy®, Chicago, IL, USA), and the values were rounded up to the nearest millimeter. The average score for whole-mouth PD, CAL, GI, PI, and BOP, divided by the total number of sites per mouth and multiplied by 100, was calculated for each individual.

### Sample collection, processing, and next generation sequencing

The saliva samples were obtained after 4–6 weeks after the initial biopsy of the lesion. Before sampling, participants were instructed to rinse their mouths with 0.9% saline solution. Five ml of unstimulated saliva were collected, placed in sterile 50-ml containers, and transferred to the laboratory promptly. DNA/RNA Shield reagent (Zymo Research Corp, CA, USA) was added for stabilization and stored at -80^o^C until the nucleic acid extraction. DNA was extracted using Quick-DNA Fecal/Soil Microbe Kits (Zymo Research Corp, CA, USA), according to the manufacturer’s instructions. The concentration and purity of DNA samples were determined with Qubit (Thermo Fisher Scientific, Waltham, MA, USA) before sequencing.

Next-generation sequencing was performed by targeting V3-V4 gene regions of the 16 S rRNA. 16 S Universal Eubacterial primers (16 S Forward: TCG TCG GCA GCG TCA GAT GTG TAT AAG AGA CAG CCT ACG GGN GGC WGC AG; 16 S Forward Reverse: GTC TCG TGG GCT CGG AGA TGT GTA TAA GAG ACA GGA CTA CHV GGG TAT CTA ATC C) were used for amplification [21, 22]. 2-step PCR was used in the library preparation process. Using KAPA HiFi HotStart ReadyMix, 25 cycles of PCR were carried out individually (Roche Diagnostics GmBH, Mannheim, Germany). In the first PCR step, the PCR condition was 3 min at 95 °C, then 30 s at 95 °C, 30 s at 55 °C, 30 s at 72 °C for 25 cycles, and finally, a single cycle at 72 °C for 5 min. In the second PCR application, Nextera XT Index Primer 1 and Nextera XT Index Primer 2 sets (Illumina, CA, USA) were added to the Illumina index and adapter sequences. In this PCR step, the PCR condition was 3 min at 95 °C; 30 s at 95 °C, 30 s at 55 °C, 30 s at 72 °C for eight cycles, and then a single cycle at 72 °C for 5 min. The Agencourt AMPure XP kit (Beckman Coulter) was used to purify the amplicon products following both PCR cycles. PCR products were examined for band presence and relative band intensities on a 2% agarose gel following the PCR procedures. The prepared library was measured with a Qubit fluorometer and sequencing after normalization. Sequencing was performed on an iSeq100 instrument using an iSeq100i1 Reagent kit (Illumina, San Diego, CA, USA) per the manufacturer’s instructions [21, 23, 24].

### Bioinformatics and statistical analysis

The FastQC program was used for quality control following the sequencing application. Data quantities, read quality, GC distributions, kmer distributions, and potential adapter contaminations of each sample were analyzed in light of the quality control results. After quality control, reads with poor read quality (Phred Quality Score < Q20, window range of 30 bp) were excluded from all data. Low-quality base reads possible adapter contaminants, and chimeric sequences at the read tips were trimmed based on the Genomes OnLine Database (GOLD) and with the Trimmomatic tool. Reads were aligned to target organisms for taxonomic characterization using the SILVA database [21, 25]. OTU groups in each sample were identified after alignment. Data reporting, statistical analysis, and visualization were done using the R program and scripts.

## Results

### Demographic characteristics and oral health of study groups

The oral cancer lesions from patients were all squamous cell carcinoma. There were no differences in gender and age distribution between healthy individuals and patients with oral cavity cancers (Table 1). History of smoking, alcohol consumption, and smoking habits were also not significantly different between groups. Family history of cancer and educational level significantly differed between patients and controls (*p* < 0.001). All healthy controls reported more than 8 years of education (*p* < 0.001).

**Table 1:** Socio-demographic, behavioral, and comorbidity characteristics of study participants. (SD: Standard Deviation; Med: Median; Min: Minimum; Max: Maximum)

Characteristics	Healthy Controls	Oral Cavity Cancer Patients	*p*-value
	*N*(%)	*N*(%)	
**Gender**			0.6911
*Male*	6(50)	6(60)	
*Female*	6(50)	4(40)	
**Age (years)**			0.1872
*mean* **±** *SD*	53.5 ± 13.9	63 ± 13.1	
*Med(min-max)*	57(28–71)	61.5(46–89)	
**Marital Status**			0.6241
*Married*	10(83.3)	7(70)	
*Not married*	2(16.7)	3(30)	
**Education**			< **0.001**
≤ 8 years	0(0)	6(60)	
> 8 years	12(100)	4(40)	
**BMI**			0.3562
*mean* **±** *SD*	23.4 ± 3.6	25.02 ± 4.88	
*Med(min-max)*	22.7(18.38–32.05)	25.27(18.69–34.75)	
**Comorbidities**			1
*No*	10(83.3)	7(77.8)	
*Yes*	2(16.7)	2(22.2)	
**Family history of cancer**			< **0.001**
*No*	12(100)	1(10)	
*Yes*	0	9(90)	
**History of smoking**			0.096
*No*	4(33.3)	0(0)	
*Yes*	8(66.7)	10(100)	
**Current smoking habit**			1
*No*	8(66.7)	7(70)	
*Yes*	4(33.3)	3(30)	
**Alcohol consumption**			0.646
*No*	8(66.7)	8(80)	
*Yes*	4(33.3)	2(20)	
**Red meat consumption (days per week)**			0.619
*mean* **±** *SD*	4.4 ± 1.0	4.7 ± 1.6	
*Med(min-max)*	5(3–6)	5(2–7)	
**Vegetable consumption (days per week)**			0.886
*mean* **±** *SD*	4.5 ± 1.0	4.7 ± 1.1	
*Med(min-max)*	5(2–6)	5(3–7)	
**Fruit consumption**			0.489
**(days per week)**			
*mean* **±** *SD*	4.9 ± 0.9	5.3 ± 1.4	
*Med(min-max)*	5(4–7)	5(3–7)	

As shown in Table 2, all the patients in this study rated their oral health as good. Oral cavity cancer patients perceived more signs of gingival bleeding and gum swelling and redness (*p* < 0.001). Healthy controls performed more tooth-brushing (*p* < 0.003) and used dental floss/interdental brush (*p* < 0.005) than oral cavity cancer patients. All oral cavity cancer patients reported a history of periodontal disease (*p* < 0.005).

**Table 2:** Self-reported oral health and oral hygiene habits of the participants (^1^Fisher Exact test, ^2^Mann Whitney U test, SD: Standard Deviation; Med: Median; Min: Minimum; Max: Maximum)

	Healthy Controls *N*(%)	Oral Cavity Cancer Patients *N*(%)	*p*
Self-rated oral health			-
*Good*	12(100)	10(100)	
Self-reported gingival bleeding			**< 0.001** ^**1**^
*No*	11(91.7)	0	
*Yes*	1(8.3)	10(100)	
Self-reported gum swelling and redness			**< 0.001** ^**1**^
*No*	11(91.7)	0	
*Yes*	1(8.3)	10(100)	
Having dental check-up			0.084^2^
*No*	3(25)	7(70)	
*Yes*	9(75)	3(30)	
Tooth brushing			**0.003** ^**2**^
*No*	0	6(60)	
*Yes*	12(100)	4(40)	
Using dental flossing/interdental brush			**0.005** ^**1**^
*No*	5(41.7)	10(100)	
*Yes*	7(58.3)	0	
History of periodontal diseases			**0.005** ^**1**^
*No*	7(58.3)	0	
*Yes*	5(41.7)	10(100)	
Having dental prosthesis			0.195^2^
*No*	12(100)	8(80)	
*Yes*	0	2(20)	

Direct assessment of oral health demonstrated that the patients with oral cavity cancers suffered from poor oral health (higher PI, GI, BOP%, PD, CAL, GR) in comparison to healthy controls (Table 3, *p* < 0.001). Oral examination revealed that all the oral cavity cancer patients had swelling and gingival bleeding. All patients had an active or a history of periodontal disease, and 2 of 10 wore partial removable dental prostheses.

**Table 3:** Clinical assessment of oral health in study participants (^1^Fisher Exact test, ^2^Mann Whitney U test, SD: Standard Deviation; Med: Median; Min: Minimum; Max: Maximum)

	Healthy Controls *N*(%)	Oral Cavity Cancer Patients *N*(%)	*p*
Gingival Index (GI)			**< 0.001** ^**3**^
*mean* ± *SD*	0.6 ± 0.1	1.8 ± 0.4	
*Med(min-max)*	0.6(0.5–0.9)	1.7(1.2–2.4)	
Plaque Index (PI)			**< 0.001** ^**3**^
*mean* ± *SD*	0.3 ± 0.1	2 ± 0.3	
*Med(min-max)*	0.4(0.02–0.5)	1.9(1.6–2.7)	
Bleeding on probing(BOP)			**< 0.001** ^**3**^
*mean* ± *SD*	3.3 ± 1.6	100 ± 0	
*Med(min-max)*	3.3(0–6)	100(100–100)	
Probing Depth (PD, mm)			**< 0.001** ^**3**^
*mean* ± *SD*	1.4 ± 0.1	3.6 ± 0.4	
*Med(min-max)*	1.5(0.3–1.5)	3.5(3.2–4.3)	
Clinical attachment level (CAL, mm)			**< 0.001** ^**3**^
*mean* ± *SD*	1.4 ± 0.3	4.7 ± 0.4	
*Med(min-max)*	1.5(0.3–1.5)	4.6(4.2–5.5)	
Gingival recession (GR, mm)			**< 0.001** ^**3**^
*mean* ± *SD*	0 ± 0	1.1 ± 0.7	
*Med(min-max)*	0(0–0)	1.1(0.1-2.0)	

### Microbiological findings in oral cancer patients

**Fig. 1:**
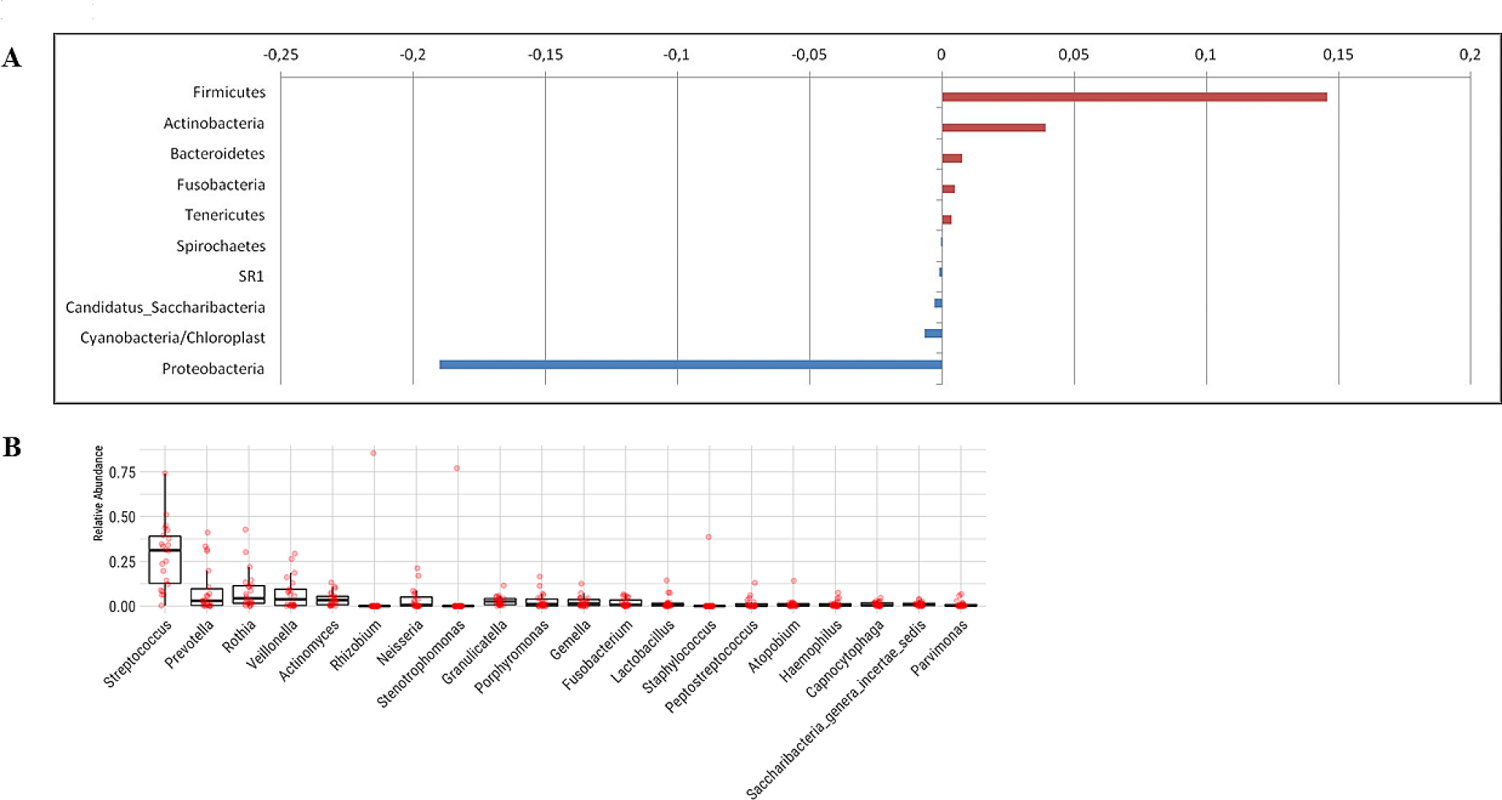
The most abundant bacterial phyla and genus. Panel **A** shows the alterations in the most abundant 10 bacterial phyla in oral cavity cancer and control groups. Panel **B** demonstrates the most abundant 20 bacterial species in all samples

**Fig. 2:**
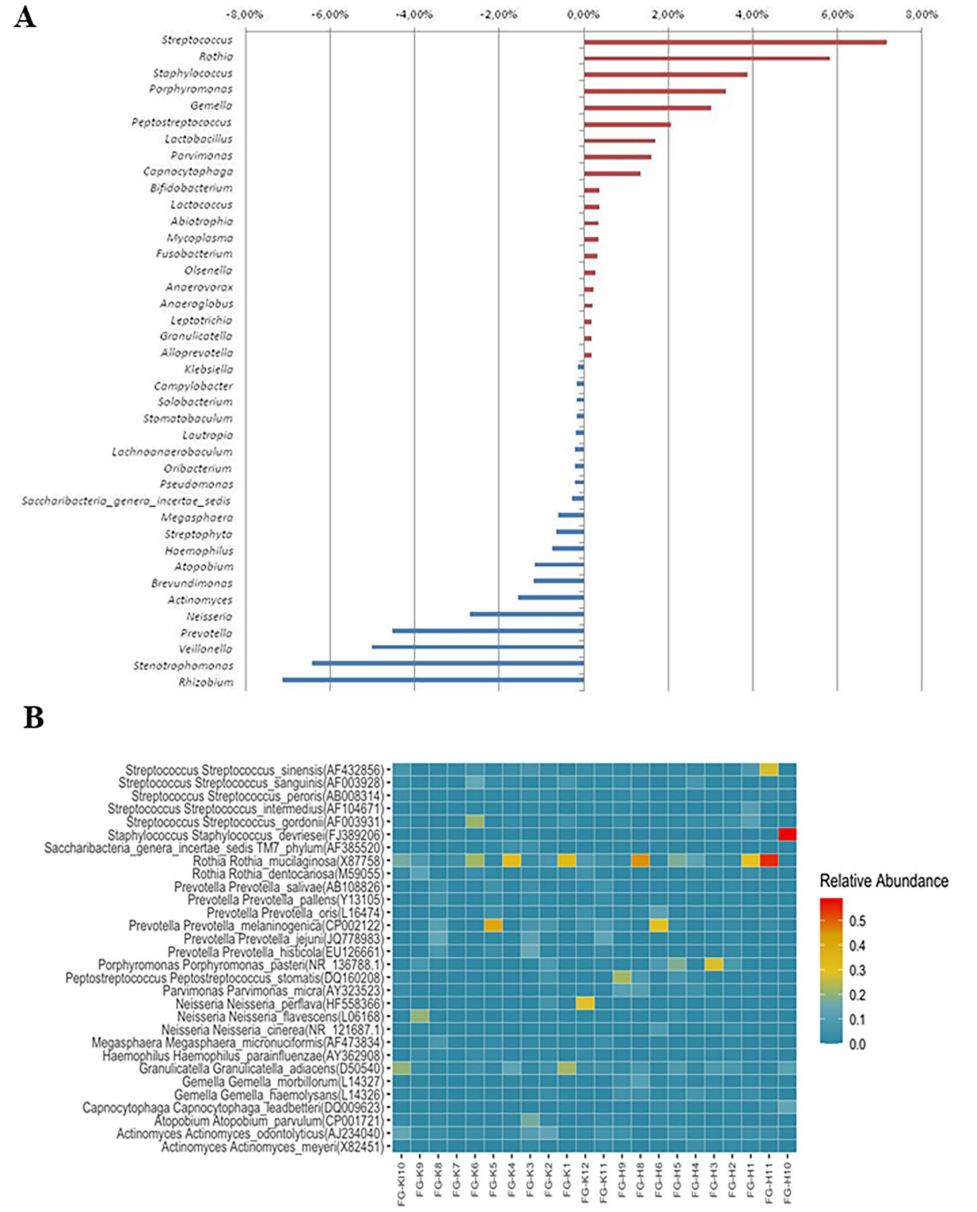
The most prevalent bacterial genus and species. Panel **A** demonstrates the changes in the amount of the most abundant 40 bacterial genera in oral cavity cancer patients and the control groups. Panel **B** shows the first 30 species with the highest incidence according to metagenomic analysis obtained from both oral cavity cancer patients and the controls in each sample (H: Patients, C: Control)

Figure 1 demonstrates the most abundant bacterial phyla and genera and the alterations of the abundances in these taxa among oral cavity cancer patients and healthy controls. Figure 2 shows the most prevalent bacterial genus and species in oral cancer patients and healthy controls.

There were 31 phyla in total, with 5 of these dominating in all samples: *Firmicutes, Proteobacteria, Bacteroidetes, Actinobacteria*, and *Fusobacteria*, with the remaining 26 phyla having a relative abundance of less than 1%. In the control samples, there was a dominance of *Firmicutes* (44.9%), *Bacteroidetes* (13.5%), *Proteobacteria* (23.1%), *Actinobacteria* (13.3%), and *Fusobacteria* (2.7%). In contrast, in the oral cavity cancer samples, *Proteobacteria* (4.1%) was decreased, *Firmicutes* (59.2%), *Bacteroidetes* (14.2%), *Actinobacteria* (17.2%) and *Fusobacteria* (3.2%) were elevated. *Cyanobacteria* increased approximately eight times in the cancer group compared to the healthy control group. *Aggregatibacter, Haemophilus*, and *Neisseria*, which are the members of *Proteobacteria*, decreased in the patient group in parallel with the total decrease in the phylum. *Fusobacterium*, a member of the phylum of *Fusobacteria, Lactobacillus*, and *Gemella*, a member of *Firmicutes*, increased in the patient group in parallel with the phylum. The bacterial genus with the highest rate in the patient and control groups was *Streptococcus*, followed by *Rothia, Prevotella*, and *Veionella*. While *Streptococcus* was found in 25% of the healthy controls, 32% of the patients with oral cancer had *Streptococcus*. While the *Rothia* was 6% in the controls, its presence doubled (12%) in the patient group. Likewise, *Peptosteptococcus*, *Tannerella*, and *Lactobacillus* were higher in the oral cavity cancer group. *Prevotella* was detected in 11% of controls, while only 7% of the patients were positive. *Veillonella* was found at an average rate of 9% in the controls; its presence decreased to 4% in the patient group.

**Fig. 3:**
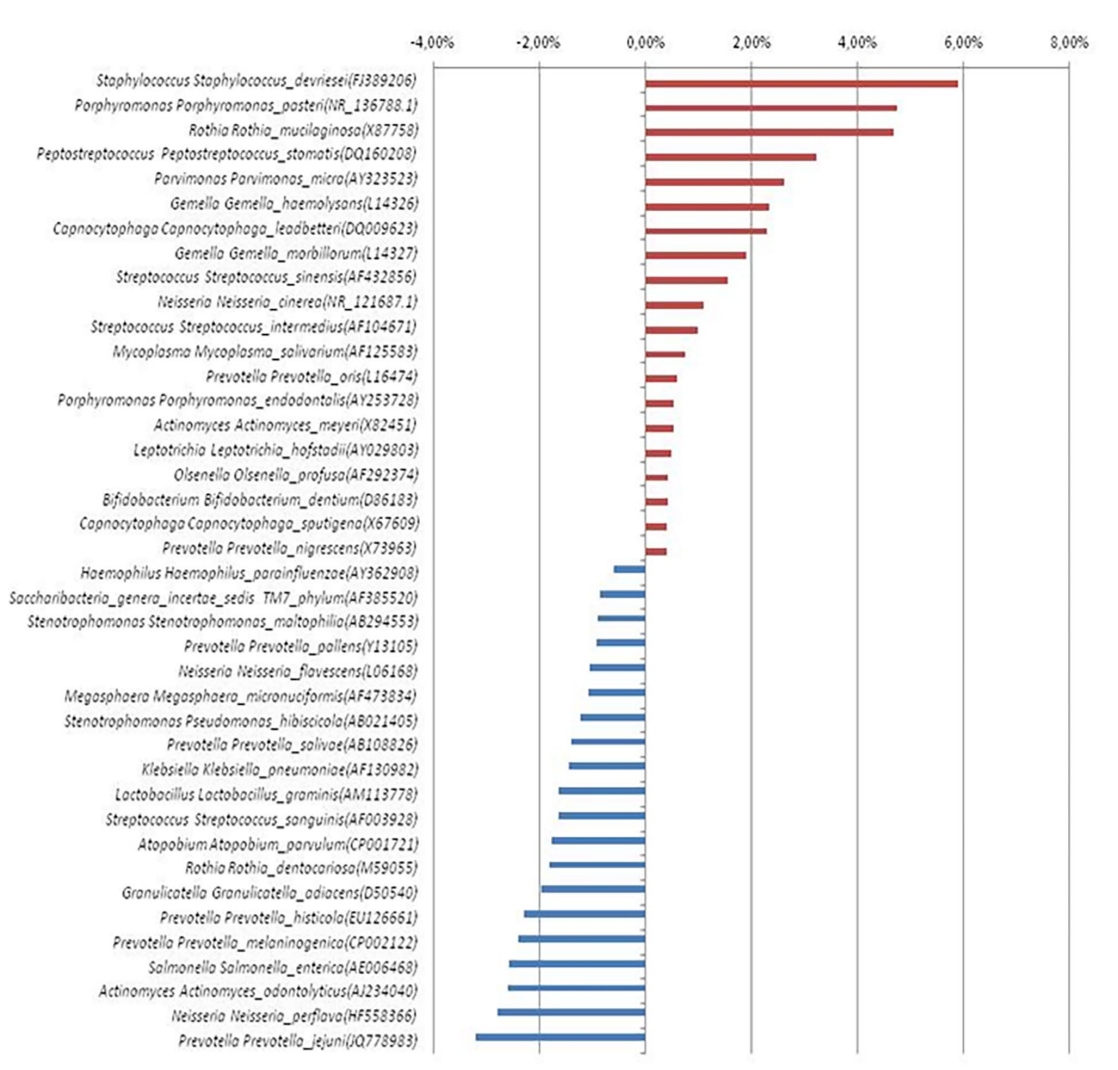
The most abundant 40 bacterial species (OTU) in oral cavity cancer patients and the control groups

At the species level, *Porphyromonas gingivalis* increased 6.5 times in oral cavity cancer patients. *Gemella haemolysans* was significantly higher in the oral cancer patients, while *Corynebacterium durum, Lachnoanaerobaculum umeaens, Neisseria subflava, Actinomyces massiliensis, and Veillonella dispar* were significantly lower (*p* < 0.05). Alterations in the amount of the most abundant 40 bacterial species in oral cavity cancer patients and the control groups are shown in Fig. 3. *F. nucleatum* was elevated 2.8-fold, and *A. actinomycetemcomitans* declined 1.3-fold compared to the healthy controls.

### Impact of smoking and alcohol consumption on oral microbiota in oral cancer

**Fig. 4:**
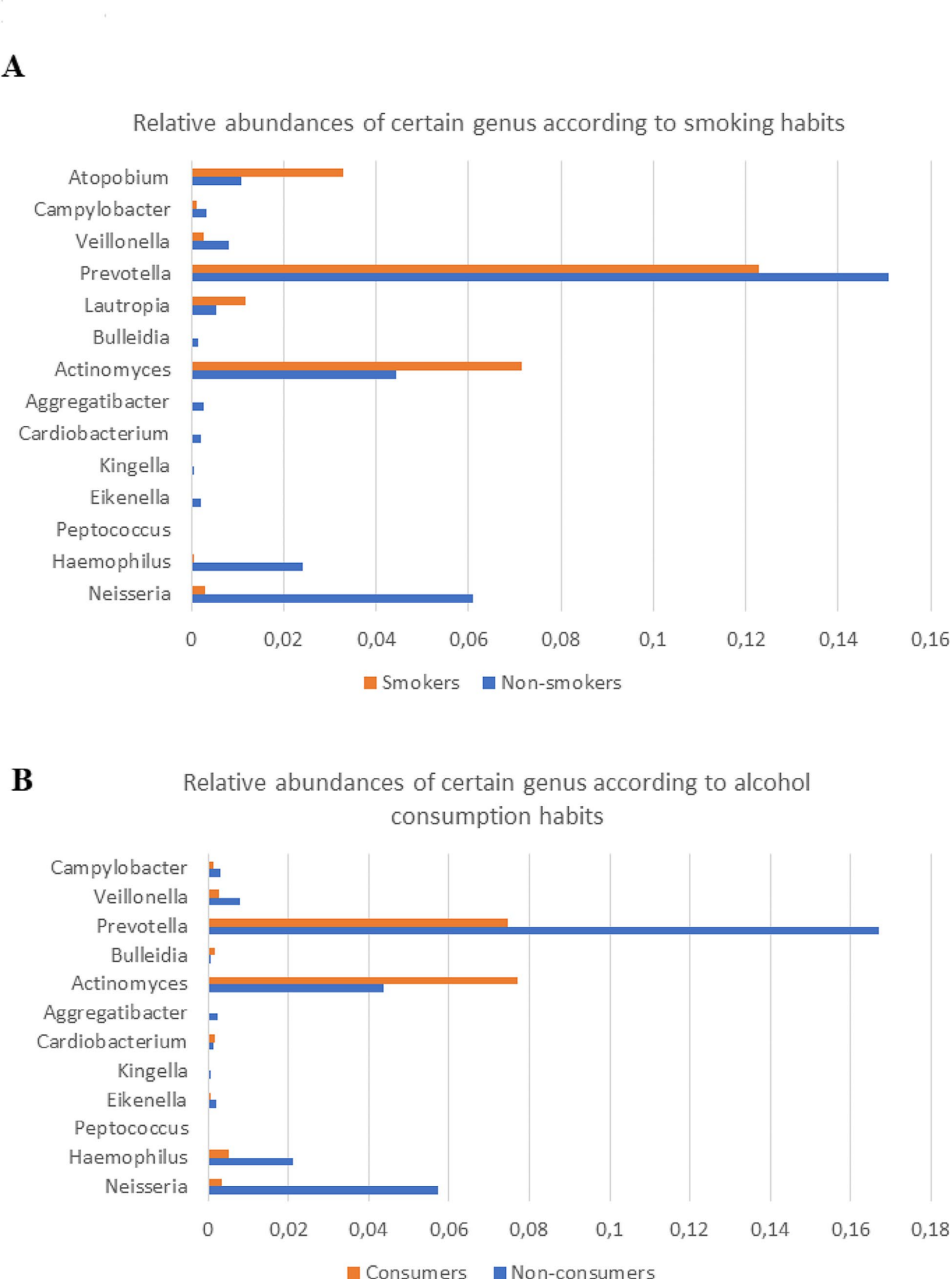
The effects of alcohol consumption and smoking habits on oral microbiota. Panel **A** shows the variation in the relative abundances of certain species among smokers and non-smokers. Panel **B** shows these alterations due to alcohol consumption

The effects of smoking habits and alcohol consumption on oral microbiota are shown in Fig. 4. The data for abundances were given as supplementary tables. At the genus level, while *Neisseria, Haemophilus, Peptococcus, Eikenella, Kingella, Cardiobacterium, Aggregatibacter, Prevotella, Veillonella, Campylobacte*r, and *Bulleidia* were lower, *Actinomyces, Atopobium and Lautropia* were increased in smokers. At the species level, the abundance of *Haemophilus parainfluenzae, Saccharibacteria genera incertae sedis, Rikenellaceae bacterium, Gemella palaticanis, Rothia aeria, Prevotella saccharolytica, Prevotella maculosa, Fusobacterium periodonticum, Prevotella bryantii, Porphyromonas pasteri, Prevotella shahii, Treponema socranskii, Cardiobacterium hominis, Aggregatibacter aphrophilus, Dialister pneumosintes, Veillonella rogosae, Haemophilus sputorum, Neisseria elongata* species were found to be significantly lower in smokers (*p* < 0.05).

Alcohol consumption changed the oral microbiota in oral cancer. At the genus level, *Neisseria, Haemophilus, Eikenella, Kingella, Aggregatibacter, Prevotella, Veillonella*, and *Campylobacte*r were lower, while *Actinomyces, Bulleidia, and Cardiobacterium* were more abundant in alcohol consumers. In particular, members of the *Proteobacteria* family, *Aggregatibacter* was 67-fold lower, *Neisseria* was 15-fold lower, and *Haemophilus* was 4-fold lower in alcohol consumers. At the species level, *Fusobacterium periodonticum, Prevotella bryantii, Gemella palaticanis, Prevotella maculosa, Porphyromonas pasteri, Prevotella scopos, Aggregatibacter aphrophilus, Kingella oralis, Prevotella veroralis*, and *Haemophilus sputorum* were significantly higher in non-consumers compared to alcohol consumers (*p* < 0.05).

### Impact of oral care on oral microbiota in oral cancer

**Fig. 5:**
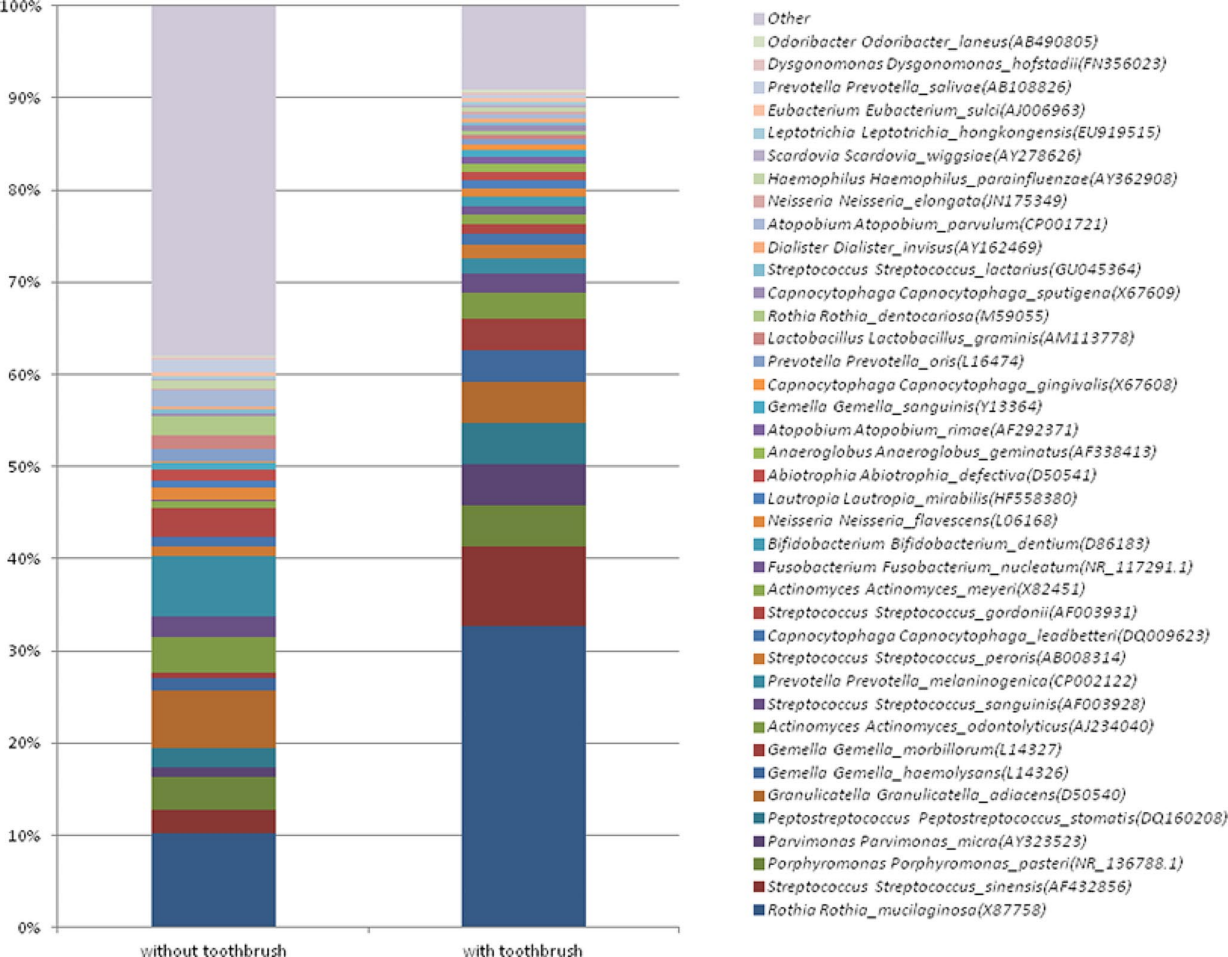
The effect of daily brushing habits on the relative abundance of the bacterial species obtained from oral cavity cancer patients

As shown in Fig. 5, the most abundant 10 species in oral cavity cancer patients who have daily tooth-brushing habits were (in ranking order) *Rothia mucilaginosa, Streptococcus sinensis, Porphyromonas pasteri, Parvimonas micra, Peptostreptococcus stomatis, Granulicatella adiacens, Gemella haemolysans, Gemella morbillorum, Actinomyces odontolyticus, Streptococcus sanguinis*, respectively. In patients who did not have daily tooth-brushing habits, the most abundant 10 species were *Rothia mucilaginosa, Prevotella melaninogenica, Granulicatella adiacens, Actinomyces odontolyticus, Porphyromonas pasteri, Staphylococcus devriesei, Streptococcus gordonii, Streptococcus sinensis, Prevotella jejuni, Streptococcus sanguinis*, respectively. In brushing and non-brushing patients with oral cancer, *Rothia mucilaginosa* was the most abundant species; it was 3.2-fold higher in participants with daily tooth brushing habits. In patients with daily oral care, *Gemella morbillorum, Parvimonas micra, Gemella haemolysans, and Peptostreptococcus stomatis* levels were increased by 6, 4, 2.6, and 2.3 fold, respectively. *Staphylococcus devriesei* and *Prevotella jejuni* were 10.000- and 100-fold higher, respectively, in patients who did not have daily tooth brushing habits. *Prevotella melaninogenica, Streptococcus sinensis, and Streptococcus gordonii* were increased at the species level.

Major periodontopathogens, *Porphyromonas gingivalis, Treponema denticola, and Tannerella forsythia*, were detected 70, 4, and 2-fold higher, respectively, in those who did not have daily tooth-brushing habits, suggesting a periodontal disease-associated shift in dysbiosis in oral cancer. Another major periodontopathogen, *Aggregatibacter actinomycetemcomitans*, was also not detected in those who brush their teeth daily. Among orange complex bacteria, *Fusobacterium nucleatum* was found 4 times less in those who brush their teeth daily compared to those who do not brush their teeth daily. *Prevotella intermedia* was not detected in those who brush their teeth daily; the levels of *Lachnoanaerobaculum umeaense, Prevotella bryantii, Fretibacterium fastidiosum, Porphyromonas endodontalis, Prevotella shahii, Catonella morbi, Prevotella veroralis, Porphyromonas catoniae, Prevotella pallens*, and *Saccharibacteria genera incertae sedis* were found significantly lower. In contrast, *Rothia amarae* levels were significantly higher in those with daily tooth brushing habits than those without. While the levels of *Corynebacterium durum*, *Neisseria subflava*, and *Lachnoanaerobaculum umeaense* were significantly higher in individuals using partial dental prosthesis compared to dentate individuals, *Gemella haemolysans* was significantly lower (*p* < 0.05).

## Discussion

This study was designed to test the hypothesis that oral microbiota dysbiosis was associated with oral cavity cancer. In patients with primary oral cavity cancer who met the inclusion and exclusion criteria, oral health data were collected, and the oral microbiome was characterized by next-generation sequencing. The results showed that the patients with oral cavity cancers had poorer oral health than healthy controls. Genus and species-specific differences supported a dysbiotic oral microbiome associated with oral cancer, with periodontopathogens dominating the samples. Collectively, these data suggested that patients with oral cancer had a distinct oral microbiome composition that is affected by personal daily habits and may be associated with the pathogenicity of the disease.

The human oral microbiome is the second-most numerous microbiota in the human body, with more than 700 species. Because the oral microbiome influences metabolic and immunological responses, bacterial dysbiosis has been linked to local and systemic illnesses, including their role in malignancies [9, 26]. Since cancer staging is crucial in oncotherapeutic planning, early detection may alter the course of treatment, survival rates, and quality of life. However, no reliable biomarkers for early diagnosing oral cavity malignancies are currently available, where detection of oral microbial shifts may also serve as predictors of oncopathogenesis. Therefore, oral microbiota composition and specific species in patients with oral cavity cancer can provide a non-invasive diagnostic technique for oral cavity cancers [3, 26].

Oral health is associated with inflammatory changes in the oral cavity. To assess the oral health of our patients with oral cancer, we used both self-reported and direct professional measures of diseases. The data demonstrated that periodontal and, in general, oral health ​​was poor in oral cancer patients compared to the healthy controls, which is in line with previous reports where 76% of cancer patients had advanced periodontal disease and periodontal pockets deeper than 6 millimeters [15, 27]. These findings support the premise that periodontal disease may be a risk factor for oral cavity cancer [6, 28–30], where poorer oral health was linked to a greater risk of cancers of the head, neck, and esophagus [31]. Several studies use self-reported measures to determine oral health status, which can be misleading [32]. In our study, oral cavity cancer patients stated that they had good oral health while they had more signs of gingival bleeding, swelling, and redness. The self-reported data, therefore, are subjective. Indeed, a study showed that all cancer patients brushed their teeth up to twice daily, but most have advanced periodontal disease [27]. To validate the self-reported data, we used specific oral health indicators such as PI, GI, BOP%, PD, CAL, and GR, which were recorded by a periodontist. This approach allowed us to accurately link oral health, oral cancer, and oral microbiota, demonstrating that poor oral health was associated with increased periodontal disease in patients with oral cancer, in line with a previous study suggesting poor oral hygiene as a predictor of oral cancer [33]. Likewise, our data support the previous findings where a history of periodontitis was associated with oral squamous cell carcinoma and increased head and neck cancer risk [34, 35].

Our data presented many novel microbial signatures associated with oral cancers where specific microbial genera may be potential markers for oral cavity malignancies. The most abundant were *Firmicutes, Proteobacteria, Bacteroidetes, Actinobacteria*, and *Fusobacteria*. While *Streptococcus* and *Rothia* increased in the cancer group, *Prevotella* and *Veillonella* decreased. Some of these findings align with previous reports where genus-level profiles showed that *Streptococcus* and *Prevotella* dominated all samples, *Lactobacillus* was abundant, and *Haemophilus, Neisseria, Gemellaceae, Rothia*, and *Aggregatibacter* decreased in saliva [3]^,^ [26]. In contrast to our findings, however, these previous studies demonstrated a decrease in the *Streptococcus* genus, possibly due to patient population-specific variations that need further study. Likewise, we demonstrated an increased abundance of *Rothia* in oral cavity cancer patients, contrasting with these previous reports [3]. .

Our data also demonstrate mechanistic and pathogenetic insights. One of our most important observations was that there were 6.5- and 2.7-fold *P. gingivalis* and *F. nucleatum* in the oral microbiota of patients with oral cavity cancer compared to controls. Since *P. gingivalis* and *F. nucleatum* can generate inflammation, cell proliferation, and cellular invasion in OSCC through several routes [36], their carcinogenic potential may be critical for oral cavity cancers through the production of interleukin-6, tumor necrosis factor-α, matrix metalloproteinases, reduced apoptosis, and regulation of the p53 tumor suppressor gene. To this end, *F. nucleatum* can specifically promote cell proliferation and increase interleukins and other MMPs that affect the progression of tumor invasion and metastasis, potentially resulting in increased oncogene and pro-inflammatory cytokine transcriptional activity [12, 37]. Indeed, *F. nucleatum* can be a risk factor for a variety of malignancies, including tumors of the oral cavity through a persistent infection, the interaction of cell surface molecules with the immune system and stromal cells, immune evasion, and immunological suppression and virulence factors such as FadA, Fap2, LPS, all of which linked to transforming epithelial cells into tumor cells [38].

Beyond association, the link between specific periodontopathogens and oral cancers is unclear. Mechanistically, inter-species associations may shed light on this puzzle where NGS can be a valuable tool to identify potential pathological consequences of bacteria-bacteria interactions. Our data in a highly defined cohort provided compelling evidence for such mechanistic insights. To this end, one of the bacteria reported to be strongly associated with the tumor site of oral cavity tumors is *Gemella haemolysans* [39]. We found that *G. haemolysans* was significantly elevated in the oral microbiota of oral cavity cancer patients compared to the healthy controls. Higher levels of *G. haemolysans* and *Gemella parahaemolysans* were recently linked to oral lichen planus, which is a risk factor for oral cancers [40]. Meanwhile, *P. gingivalis* colonization was 6.5 times higher in the oral microbiota of cancer patients compared to the healthy controls in this study. The underlying mechanism for *P. gingivalis*, despite being regarded as a significant etiological agent in oral cavity malignancies, is unknown [41]. . In contrast to the situation in cancer, *G. haemolysans* decreases the oral microbiota of periodontitis patients and inhibits *P.gingivalis* by secreting protein components in vitro [42]. In the context of oral cancers, however, the interspecies association between *G. haemolysans* and *P.gingivalis* may be reversed, suggesting a different microenvironment for microbial interactions. Metatranscriptomic analyses are required to identify these mechanistic interactions in detail.

Poor oral hygiene, poor nutrition, and wearing dental prostheses were associated with an increased risk of oral cancer in some studies [4, 6, 15]. However, the mechanism for poor oral hygiene making individuals susceptible to oral cavity cancer and the role of bacterial dysbiosis in the emergence of oral cancer has not been shown yet. Oral microbiota analysis revealed that *Rothia mucilaginosa* is the most abundant bacterial species found in oral cavity cancer patients regardless of their daily tooth brushing habits. *R. mucilaginosa* was found to be 3.2-fold less in the ones who do not brush their teeth daily, which is in line with previous studies [3]^,^ [43], suggesting that tooth brushing may play a role in preventing the severity of oral cavity cancers. Since *Rothia* spp. is known to produce enterobactin, which is the most potent iron-binding siderophore known among the siderophores, this finding may provide therapeutic insights of adding siderophore molecules to the cancer treatment to reduce cancer cell access to the iron molecules [44, 45].

Our results revealed the enrichment of *P. jejuni* and *P. melaninogenica* in the participants with poor oral hygiene habits, suggesting the potential contribution of these species to oral cavity cancer in line with a previous report [46]. In addition to such species, we defined novel bacteria in patients with oral cancer. For example, *Staphylococcus devriesei* was 10.000-fold higher in patients who did not have daily tooth brushing habits. This *Staphylococcus* species was described in 2010 in teat apices and milk of dairy cows [47] and from cases of bovine IMIs in South Africa in 2018 [48]. Ours is the first study that identified *S. devriesei* from a human specimen, suggesting a potential link to oral health in oral cancer.

Smoking and alcohol are independent risk factors for head and neck malignancies and carcinogens that cause DNA damage and incorrect DNA repair ^24^. Additionally, their impact on changing the oral microbiome was previously reported, implying that smoking facilitated the early acquisition and colonization of pathogens in oral biofilms [49]^,^ [50]^,^ [51]. However, the link between smoking and oral microbiome in oral cancers is not that clear. We found that *Neisseria, Haemophilus, Peptococcus, Eikenella, Kingella, Cardiobacterium, Lautropia, Prevotella, Veillonella, Campylobacter, Bulleidia*, and *Aggregatibacter* decreased, and *Actinomyces* and *Atopobium* increased in smokers. In particular, *Aggregatibacter* and *Haemophilus* decreased 100 and 50-fold in smokers, respectively. At the species level, *Rothia aeria* and *Haemophilus parainfluenzae* were significantly reduced in smokers. *Fusobacterium periodonticum* and *Porphyromonas pasteri*, associated with a healthy oral microbiome, were significantly lower in smokers (*p* < 0.05) [52]. *Haemophilus parainfluenzae, Haemophilus sputorum, and Neisseria elongata*, which are members of *Proteobacteria*, were significantly lower in the samples obtained from smokers. Lower levels of *Proteobacteria* were associated with the development of oral cancers, collectively suggesting that smoking, a key risk factor for many cancers, may affect the levels of some species, resulting in the development of oral cancer.

Alcohol consumption was also positively associated with dysbiosis in oral cancers in parallel with the decrease of the members of the oral microbiota, such as *Neisseria* and *Haemophilus*, in infections that seem to affect the immune system, such as COVID-19 [53]. A similar shift was seen for *Lactobacillales*, *Actinomyces, Leptotrichia*, and *Cardiobacterium-* the genera enriched in people who consumed more alcohol. This also provides an important mechanistic insight because *Neisseria* can convert ethanol into the human carcinogen acetaldehyde, suggesting high *Neisseria* levels may be associated with carcinogenesis [54]. A recent study revealed a negative correlation between the abundance of *Neisseria* in saliva and ACH production. Even though *Neisseria* species are the main producers of ACH in vitro, the salivary microbiota profile with a lower relative abundance of these species was independently linked to high ACH production ability, demonstrating the importance of the interplay of oral microbiome [52]. In our study, *Actinomyces* and *Cardiobacterium* levels increased from %4 to %7 in alcohol consumers, similar to a previous work [54]. However, *Neisseria* levels decreased 15-fold with alcohol consumption. In addition to *Neisseria*, alcohol consumers had 67 times lower *Aggregatibacter* levels and 4 times lower *Haemophilus* levels, which are members of *Proteobacteria* as well.

The sample size was the main limitation of our study. However, this was due to strict inclusion and exclusion criteria, which allowed us to determine significant and highly relevant alterations of oral microbiota in cancer cases in a well-defined cohort. As opposed to other association studies, oral cancers cannot be longitudinally assessed by definition as they are removed upon diagnosis. In addition, there is a paucity of knowledge in this field where studies examining the oral microbiome in oral cancer patients are limited. Needless to add, it is not trivial to characterize oral cancer patients, collect samples, and perform next-generation sequencing, which is one of the key reasons these studies are limited. Therefore, our work addressed this gap in knowledge. More controls can be added to dissect the role of periodontitis and other forms of periodontal disease in the pathogenesis of oral cancers. We, therefore, refrained from making any bold statements suggesting such a link since periodontal disease not only includes microbial but also inflammatory factors that may change the course of oral cancers. What was measured in our work was the collective association of the entire oral microbiome with the presence of oral cancer. Therefore, this observation overrides the site-specific impact of periodontal inflammation. Nevertheless, our study demonstrated that several microbial species, including major microorganisms that are well-linked to periodontitis, were also associated with oral cancers. We intentionally did not choose to compare our patients in this study to a group of patients with periodontitis because the goal of the study was not to elucidate the role of periodontitis. Our unbiased approach allowed us to identify microbial species that are associated with oral cancers compared to those without any oral cancer. Since none of the control subjects had periodontal diseases (as mentioned in Table 2), we demonstrated a clear association between oral cancers and specific microbial species of the oral cavity, some of which were linked to periodontal disease. The results demonstrated that even in the absence of periodontitis, bacterial species that play a role in periodontal disease were associated with oral cancer, which posits a fascinating question: Are periodontopathogens only limited to periodontal diseases in their impact, or do their effects go beyond the cause and severity of periodontitis? While this question was beyond the scope of this work, our ongoing studies will be focused on this topic. Whether dysbiosis is a cause or consequence of the malignancy is yet to be determined; however, the interspecies associations that we demonstrated are critical for understanding the pathobiology of oral cancers and the role of oral microbiome.

## Conclusion

Our data suggested that patients with oral cancer had worse oral health conditions and a distinct oral microbiome composition that is affected by personal daily habits and may be associated with the pathogenicity of the disease. Improved oral hygiene and treatment of periodontal disease may help limit the development or spread of oral cancer. While the results prevent us from making any further mechanistic claims due to the study’s cross-sectional design, the findings provide a basis for future studies that would test the role of oral microbial dysbiosis in oral cancer.

### Electronic supplementary material

Below is the link to the electronic supplementary material.

Supplementary Material 1

## Data Availability

No datasets were generated or analysed during the current study.
